# OP26 Artificial Intelligence For Literature Screening And Selection: Does The Evidence Support Its Use In Systematic Literature Reviews?

**DOI:** 10.1017/S0266462324000898

**Published:** 2025-01-07

**Authors:** Dominika Rekowska, Sera Sahbaz Gulser, Nita Santpurkar, Grace E. Fox, Shayan Ali, Nick Halfpenny, Petra Nass

## Abstract

**Introduction:**

The past decade has seen an exponential increase in peer-reviewed clinical research literature. Consequently, preparing and updating systematic literature reviews (SLRs) is more resource intensive and costly. Artificial intelligence (AI) could potentially accelerate SLR preparation. This study presents a review of evidence evaluating the accuracy of AI methods in SLR preparation and results of a case study using DistillerSR’s AI functionality.

**Methods:**

The review was based on a search of MEDLINE, Embase, and Embase Preprints databases using title/abstract keywords and subject heading synonyms for AI, machine learning, natural language processing (NLP), and publication screening and selection. The protocol is published on PROSPERO (CRD42023452391). To supplement this review, we conducted a case study with DistillerSR’s AI tools. We applied the AI classifiers, which use NLP to learn patterns from multiple SLRs across several indications, which encompassed over 15,000 references’ titles and abstracts. We then compared those patterns with the human responses to build an AI model that can be applied to other references.

**Results:**

The search identified 2,209 records. After deduplication, the titles/abstracts of 2,200 records were screened; of these, 79 full-text records were assessed. A total of 42 records met the eligibility criteria for inclusion. The majority were case studies. The most frequently reported tools were DistillerSR AI (n=9), Abstrackr (n=6), ASReview (n=2), and LiveSTART (n=2). The evidence showed efficiency gains, but accuracy varied across studies and AI tools. Results of the case study using DistillerSR’s AI tools indicated efficiency gains with adequate accuracy but with variability across different SLRs. Inclusion and exclusion of articles were consistent with the human decisions.

**Conclusions:**

The findings of our review and case study indicated that AI can be used reliably in the screening of articles for SLRs and could improve efficiency. However, the evidence is still evolving, and additional studies are needed. There is a need for clear guidelines on the role of AI in study screening and selection for health technology assessments SLRs and submissions.

